# The Role of Activated Stromal Cells in Fibrotic Foci Formation and Reversion

**DOI:** 10.3390/cells13242064

**Published:** 2024-12-13

**Authors:** Nataliya Andreevna Basalova, Maksim Alexandrovich Vigovskiy, Vladimir Sergeevich Popov, Evgeniya Alexandrovna Lagereva, Olga Alexandrovna Grigorieva, Anastasia Yuryevna Efimenko

**Affiliations:** Centre for Regenerative Medicine, Medical Research and Educational Institute, Lomonosov Moscow State University, 119192 Moscow, Russia; vigovskiyma@my.msu.ru (M.A.V.); galiantus@gmail.com (V.S.P.); e.lagereva@gmail.com (E.A.L.); grigorievaoa@my.msu.ru (O.A.G.); efimenkoay@my.msu.ru (A.Y.E.)

**Keywords:** activated stromal cells, fibrosis, myofibroblasts, fibroblast activation protein, FAPα, CD90 (THY-1)

## Abstract

Fibrotic focus is a pivotal morphofunctional unit in developing fibrosis in various tissues. For most fibrotic diseases, including progressive forms, the foci are considered unable to remodel and contribute to the worsening of prognosis. Unfortunately, the dynamics of the fibrotic focus formation and resolution remains understudied. A number of data suggest that the key cell type for focus formation are activated stromal cells marked by fibroblast activated protein alpha (FAPα) due to their high capacity for extracellular matrix (ECM) remodeling. We evaluated the dynamics of fibrotic focus formation and the contribution of the main cell types, including FAPα+ cells, in this process using a murine model of bleomycin-induced lung fibrosis. We revealed the very early appearance of FAPα+ cells in lungs after injury and assumed their important involvement to the myofibroblast pool formation. During the first month after bleomycin administration, FAPα+ cells colocalize with CD206+ M2 macrophages. Interestingly, during the reversion stage, we unexpectedly observed the specific structured foci formed by CD90+FAPα+ cells, which we suggested calling “remodeling foci”. Our findings highlight the crucial role of activated stromal cells in fibrosis initiation, progression, and reversion and provide emerging issues regarding the novel targets for antifibrotic therapy.

## 1. Introduction

The structure and function of tissues and organs can be disrupted by various damaging agents, such as mechanical trauma, viral infection, chemical exposure, allergic and immune reactions. The outcome of reparative regeneration might be partial or complete restoration of the lost function of the specific tissue or organ. However, in adult mammals, recovery mostly occurs with the development of fibrosis [1,2]. Chronic damages lead to the formation of a microenvironment which supports and continuously activates cells that ensure repair processes [3]. The outcome of such constant activation is a pathology named progressive fibrosis—a process characterized by the active synthesis of extracellular matrix (ECM) proteins (collagens, tenascin, fibronectin, etc.) and connective tissue expansion [1]. Progressive fibrosis, once started, often leads to a gradual replacement of functional tissue with connective tissue, which results in complete dysfunction of the organ and a patient’s disability or even fatal outcome.

The morphofunctional unit of many progressive forms of fibrosis of various tissues is a fibrotic focus [4,5,6,7]. It should be noted that the formed fibrotic focus in human tissues is believed to be a consequence of the inability to remodel structure. Accumulating histological data characterize the fibrotic focus as a structure consisting of a myofibroblast core and an active fibrotic front [8]. The myofibroblast core includes the deposits of misfolded collagens I, III, IV, V, and VI, as well as fibronectin (including EDA–fibronectin), synthesized by alpha smooth muscle actin-positive (αSMA+) myofibroblasts [4,8]. Along the periphery of the myofibroblast core, the area with actively proliferating and invasive fibroblasts, the so-called activated fibroblasts, is located, involved in the expansion of the fibrotic changes [9]. Recent data indicate that the emergence of activated forms of fibroblasts, marked by fibroblast activation protein alpha (FAPα), leads to the development of fibrosis in many organs and tissues [10].

There are many experimental articles and reviews describing the stages of fibrosis development. However, most of them are based on in vitro observations or static pathological material. Only a small number of papers demonstrate the histological dynamics of fibrosis development in different tissues as well as the formation of fibrotic foci in light of the cellular interaction mechanisms. It is complicated to observe this process in humans in dynamics due to ethical restrictions. However, there are few studies describing the process of foci appearance [11] and “early” and “mature” forms of foci [12] or the trajectory of cell population differentiation using current molecular and bioinformatics approaches [13]. Data based on animal models predominantly contain general histological descriptions [14,15,16,17,18,19]. However, a detailed understanding of the dynamics of the fibrotic focus formation could provide valuable clues in the search for promising therapeutic targets and determining the optimal time of exposure to these targets. Moreover, the use of a model in which the structures of fibrotic foci are remodeling and disassembly, in contrast to the progressive forms in humans, may open up novel mechanisms associated with the reversal of pathological fibrosis. Therefore, in this study, we describe in detail the dynamics of fibrotic foci formation in a model of bleomycin-induced pulmonary fibrosis in mice. This well-established animal model represents reversible fibrosis, in which, nevertheless, stable fibrotic foci are developed, and which also largely reproduces the progressive form of idiopathic pulmonary fibrosis (IPF) [20]. In this model, the interaction of the crucial participants in focus formation like activated stromal cells (FAPα) as well as their most important partners, such as myofibroblasts, M2-type macrophages, and epithelial cells, were assessed at different time points in pulmonary tissues using immunohistochemistry.

## 2. Materials and Methods

### 2.1. Animals

For the work, male mice *C57BL6/N* (Puschino, Russia) were used; at the start of the experiment, animals were 2 months old and weighed 21.3 ± 0.8 g. Animal housing and research procedures were conducted in compliance with Directive 2010/63/EU and approved by the local bioethical committee. Magnetic resonance imaging (MRI) was conducted under inhalational anesthesia supplied by the animal oxygen concentrator (Nuvo Lite 525 oxygen concentrator, NIDEK_MEDICAL, Bremen, Germany): 2–4% isoflurane mix (Laboratorios Karizoo, S. A., Barcelona, Spain) with around 93% of oxygen (V3000 Parkland Scientific (Coral Springs, Sourh Florida, FL, USA).

Pulmonary fibrosis in mice was induced using bleomycin (Bleocin #PN0113322/01, Nippon Kayaku Co., Tokyo, Japan) at a dose of 3 U/kg (volume 30 μL/mouse) diluted in sterile phosphate-buffered saline. Bleomycin was administered once intratracheally (transoral instillation). The animals were euthanized with anesthetic overdose (i.p injection of 0.3 mL of 30 mg/mL solution of Zoletil 100 (Virbac, Carros, France)). The dynamics of fibrosis development were assessed on days 3, 7, 14, and 28, and the process of fibrosis reversal was assessed through 3, 4, and 5 months after bleomycin administration (*n* = 4–5 animals in each group). After anesthesia, the lungs were perfused using a peristaltic pump by introducing 50 mL of saline solution, after which the lungs were removed and fixed in 10% neutral formalin.

### 2.2. Magnetic Resonance Imaging

The MRI scans were conducted using a ClinScan 7 T tomograph from Bruker Biospin, MRI-compatible Model 1025 (Billerik, MA, USA). Once anesthetized with air and isoflurane mixture, animals were subjected to MRI. We used a MRI-compatible Model 1025 Small Animal Monitoring and Gating System (Small Animal Instruments, Inc., Stony Brook, NY, USA) to synchronize exposure with respiration rate. Lung imaging was performed through fat-suppressed T2-weighted turbo-spin-echo sequences with specific parameters: TR (repetition time) of 1175 ms; TE (echo time) of 55 ms; echo train length of 8; field of view of 42 × 60 mm; and base resolution of 216 × 384. We counted the percentage of damaged lung tissue with Fiji software ((Fiji is Just) ImageJ 2.0.0-rc-68/1.53t/Java 1.8.0_172 (64-bit)), as we described previously [21].

### 2.3. Histological Examination

After fixation, the lungs were mounted into paraffin blocks and cut into 1 μm-thick slides. Slides were then subjected to standard deparaffinization, staining, and mounting in synthetic polymer (Dako mountains medium, Dako, Agilent, Santa Clara, CA, USA) or Aqua-Poly/Mount (PolySciences, Warrington, PA, USA). Hematoxylin and eosin staining was used to describe the general morphology of the lung tissue. Van Gieson staining was used to evaluate the deposition of ECM. These preparations were used to assess the severity of developing fibrosis according to the Ashcroft scale [22] by two independent blinded experts.

For immunohistochemical examination, sections were unmasked by boiling at 95 °C for 20 min in citrate or Tris-EDTA (TE) buffer. To reduce background fluorescence, sections were treated with 50 mM ammonium acetate, and then non-specific binding of the secondary antibodies was blocked for 1 h using normal 10% serum of the animal donor of the secondary antibodies (Abcam, Cambridge, UK) prepared in 1% bovine serum albumin (BSA) (PanEco, Moscow, Russia), followed by the incubation in primary antibodies overnight. The list of antibodies is presented in Table 1. The detection of primary antibodies (Figure S9) was performed using secondary antibodies conjugated with a fluorescent label (Alexa plus 488 (A32731; Invitrogen, Carlsbad, CA, USA), Alexa plus 594 (A21203, A11032, Invitrogen) for 2 h at room temperature in the dark. After incubation, non-specific binding sites of rabbit antibodies were blocked with 5% rabbit serum (Service Bio, Wuhan, China), and primary labeled anti-FAPα antibodies (BS-5758R-A647,1:200, Bioss Inc., Woburn, MA, USA) were applied. Nuclei were labeled with DAPI solution (Sigma-Aldrich, St. Louis, MO, USA). Microscopic examination was performed on a Leica DM600Β microscope equipped with a Leica DFC 420× camera (Leica Microsystems GmbH, Wetzlar, Germany), using representative fields of view to obtain photographs. Image processing and analysis were performed using LasX (Leica Application Suite X 3.7.1.21655) and FiJi ((Fiji is Just) ImageJ 2.0.0-rc-68/1.53t/Java 1.8.0_172 (64-bit)) software. Image panels were assembled in the programs FiJi and GIMP (GIMP 2.10.28).

### 2.4. Statistical Analysis

Statistical analysis was conducted using the GraphPad Prism software (GraphPad Prism 8). Experimental data were expressed as median (±interquartile range). The non-parametric Mann–Whitney U test was employed for two-group comparisons. Differences were considered significant when * *p* < 0.05.

## 3. Results

### 3.1. General Histological Assessment of Fibrosis Dynamics in Lung Tissue

**Figure 1:**
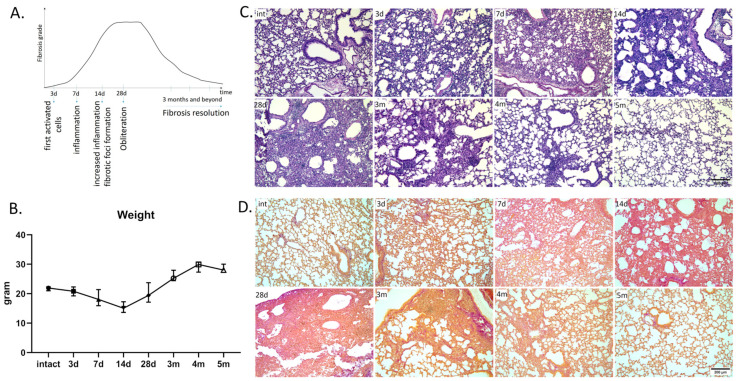
Lung fibrosis dynamics in C57BL/6 mice after single bleomycin administration. (**A**) Proposed scheme of fibrosis development and resolution. (**B**) Changes in mice weight after bleomycin administration, 3 days–5 months. Intact group (int); 3 (3d), 7 (7d), 14 (14d), 28 (28d) days and 3 (3m), 4 (4m) 5 (5m) months after bleomycin instillation. (**C**,**D**) Representative image of (**C**) hematoxylin–eosin (H&E) and (**D**) Van Gieson staining. Scale bar = 200 μm.

Based on the literature data [23,24], we selected time points for analysis corresponding to the key events in the development of progressive fibrosis (Figure 1A). Data on the timing of fibrosis reversal are very vague, so MRI analysis data were used to select the appropriate elimination period (Figure S1).

Using H&E and Van Gieson staining, a classic histology picture of developing pulmonary fibrosis over time was observed—from 0 to 1 point in intact animals to 6–7 points by Ashcroft scale in animals on the 28th day after bleomycin administration (Figure 1B–D). Thus, already at 3 days after bleomycin administration, epithelial damage and infiltration of the interalveolar septa by segmental leukocytes, macrophages, and lymphocytes along with the initial signs of compaction in the stroma were observed. In the peribronchial connective tissue, leukocyte infiltrates were found. Increase in the subpopulation of alveolar macrophages was observed on day 7. Macrophages are widely detected in the lumen of the alveoli. No later than 7 days after injury, thickening of the lung walls appeared, including perivascular and peribronchial location, providing the basis for the “early” fibrotic foci. Such foci, enriched in the ECM, had already merged by day 14 and formed obliteration fields by day 28. By 3 months after the introduction of bleomycin, some evidence of the pulmonary fibrosis reversal was observed, continuing until 5 months, when the histological pattern corresponded to the intact group of mice. It is interesting to note that at 4 months, non-standard tissue compactions were observed, dissimilar in morphology to foci and not observed in this volume and amount at earlier stages (Figure S2). 

Thus, we observed histological changes in lung tissue that corresponded to those expected, which made it possible to conduct further in-depth study of fibrosis dynamics.

### 3.2. Fibrosis Development May Begin with the Appearance of Activated Stromal Cells

As mentioned earlier, fibroblast subtypes may be the first cell types responding to tissue damage, in particular, damage of the epithelium and underlying basal membrane. Based on literature data, we hypothesized that the subtype of activated fibroblasts expressing FAPα+ is the first to respond to such damage. So, we assessed the expression of the epithelial cell marker cytokeratin and FAPα in pulmonary tissue at different time points after injury as well as localization of cells with these markers.

**Figure 2:**
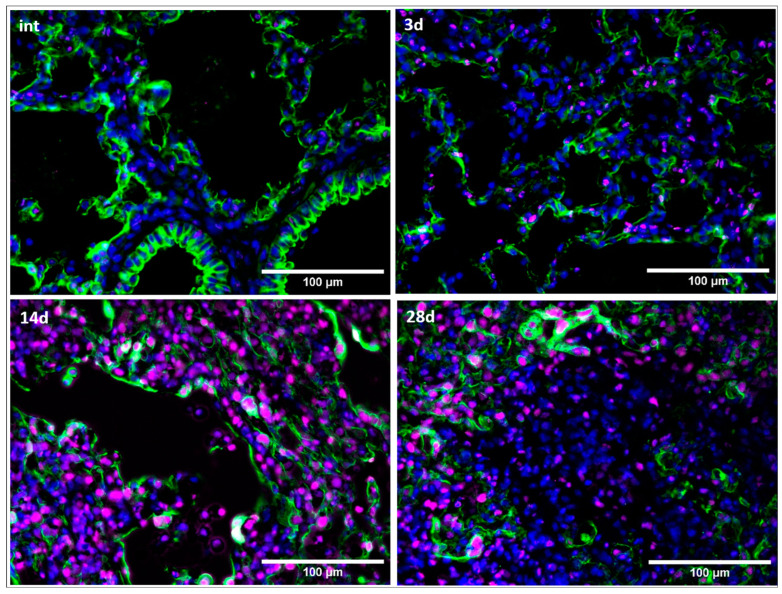
FAPα+^+^ cells appear already at the early stages of bleomycin-induced pulmonary fibrosis in mice. Representative images. Intact group (int); 3 (3d), 14 (14d), 28 (28d) days after bleomycin instillation. Pancytokeratin (green), FAPα (magenta), DAPI (blue). Scale bar = 100 μm.

We have shown that intact animals have virtually no FAPα+ cells in lungs (Figure 2 and Figure S3). However, 3 days after the administration of bleomycin, the number of FAPα+ cells increase dramatically. Initial FAPα+ cells localize in close proximity to epithelial cells (Figure 2(3d)). It is worth noting that the appearance of these cells is observed earlier than an increase in the number of CD206+ macrophages, CD90 or αSMA+ cells (Figure 4 and Figure 5). At later stages, 14–28 days after injury, a substantial increase in the number of FAPα+ cells is observed, but their localization is not associated with the epithelium. It is interesting to note that most of the areas occupied by FAPα+ cells are adjacent to the remaining lumens of the alveoli with almost obliterated fibrosis on day 28 (Figure S4). Thus, we demonstrated that FAPα+ cells appear rapidly after injury and may presumably serve as initiators of the fibrotic response.

### 3.3. Hyperproliferation of Activated Cells May Be the Root Cause of the Rapid Growth of Connective Tissue

After the initiation of fibrosis, connective tissue rapidly increases in volume, replacing the functional tissue of the organ. One of the main reasons for the rapid replacement is the hyperproliferation of stromal cells. We suggest that FAPα+ cells, belonging to the activated stroma and already appearing at early stages of fibrosis development, may be the cell subtype responsible for hyperproliferation.

**Figure 3:**
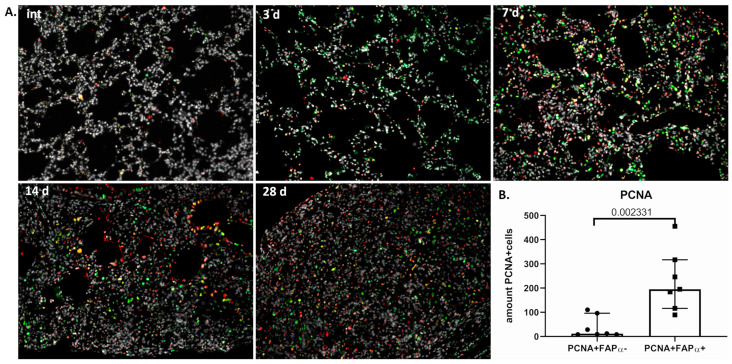
The majority of PCNA+ proliferating cells are FAPα^+^ during the development of bleomycin-induced pulmonary fibrosis in mice. (**A**) Representative image; intact group (int) 3 (3d), 7 (7d), 14 (14d), and 28 (28d) days after bleomycin instillation. PCNA (green), FAPα (red), DAPI (gray). Scale bar = 100 μm. (**B**) Quantification of PCNA^+^FAPα^−^ and PCNA+FAPα+ cells in pulmonary tissue 14 and 28 days after bleomycin injury. Data present as median (± interquartile range).

Indeed, the first single PCNA+ cells in the stroma do not appear later than the 7th day after the administration of bleomycin (Figure 3A and Figure S3). The overwhelming majority (87.2 ± 10.5%) of PCNA+ cells are double-positive FAPα+PCNA+ (Figure 3B). We observed the highest peak in the number of proliferating cells, including double-positive ones, occurring on the 14th day after the administration of bleomycin. At this time point, the double-positive cells formed compacted structures morphologically similar to fibrotic foci. However, by day 28, such cells were found only on the periphery of obliterating fibrosis zones and were practically not found at the remodeling stages.

Thus, the proliferation of FAPα+ cells can actually participate in the expansion of connective tissue. 

### 3.4. Connective Tissue Expansion in Pulmonary Fibrosis Is Due to Myofibroblast Differentiation and ECM Deposition

**Figure 4:**
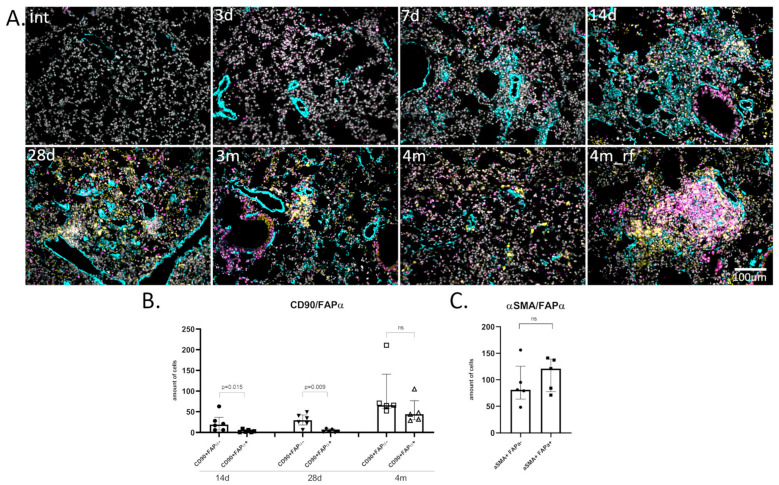
Dynamic of fibrotic foci formation and remodeling in the model of bleomycin-induced pulmonary fibrosis in mice. (**A**) Representative image; intact group (int); 3 (3d), 7 (7d), 14 (14d), 28 (28d) days, and 3 (3m) or 4 months (4m; 4m_rf—remodeling foci) after bleomycin instillation. αSMA (cyan), CD90 (yellow), FAPα (magenta), DAPI (gray). Scale bar = 100 μm. (**B**) Quantification of CD90+FAPα− and CD90+FAPα+ cells in pulmonary tissue. (**C**) Quantification of αSMA+FAPα− and αSMA^+^FAPα^+^ cells in pulmonary tissue; 14 days after bleomycin injury. Data present as median (± interquartile range), ns—non-significant.

Another reason for the overgrowth of connective tissue is certainly the appearance of cells capable of the active deposition of ECM proteins, mostly myofibroblasts. We analyzed the expression and colocalization of the stromal cell marker CD90 (THY-1), the myofibroblast marker αSMA, and FAPα in lung tissue at different time points after injury (Figure 4 and Figure S5).

#### 3.4.1. CD90/FAPα

Intact mice practically lacked the CD90 marker in lungs, with the exception of some dense areas. Small distinct areas occupied by CD90+ cells only appeared by day 7 and were localized either along the periphery of blood vessels or in areas of fibrotic foci rich in αSMA+ cells (Figure 4A(7d) and Figure S5(7d)). Interestingly, cells expressing CD90 esentially did not colocalize with αSMA+ cells. By day 28, CD90+ cells also occupied the periphery of αSMA-rich foci, or they formed new structures that have not been described in the literature before and represented highly cellular formations, which we will further call “remodeling foci” (Figure 4A(28d,4m_rf) and Figure S5(14d,14d_rf,28d,4m_rf)).

Regarding the long-term monitoring of pulmonary fibrosis in mice, from 3 months after injury the number of CD90+ cells significantly decreased, and “remodeling foci” were almost not observed (Figure 4(3m) and Figure S5(3m)). Unexpectedly, despite the higher “restoration” of the tissue at 4 months compared to 3 months, at this time point, such structures again appeared in large numbers in all studied animals, often adjacent to areas rich in FAPα (Figure 4(4m vs. 4m_rf) and Figure S5(14d, 4m vs. 4m_rf)). Moreover, if at previous stages the colocalization of FAPα and CD90 was rare, now a significant number of double-positive cells appeared in fibrotic lungs (Figure 4B, 13.6% ± 2.4% on 14 day, 12.9% ± 5.1% on 28 day vs. 36.6% ± 6.9% on 4 month). It can be assumed that these cells make a significant contribution to the remodeling of connective tissue.

#### 3.4.2. αSMA|FAPα

**Figure 5:**
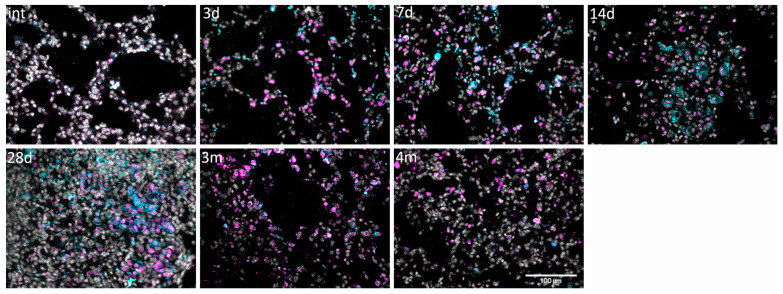
Areas occupied by CD206+ macrophages and FAPα^+^ cells are often adjacent to each other. Representative images. Intact group (int); 3 (3d), 7 (7d), 14 (14d), 28 (28d) days, and 3 (3m) or 4 (4m) months after bleomycin instillation. CD206 (cyan), FAPα (magenta), DAPI (gray). Scale bar = 100 μm.

Single αSMA+ cells could be seen in the lungs 3 days after bleomycin administration (most of them are observed to be subpleural in colocalization with FAPα). However, small perivascular “early foci” with αSMA and FAPα or simply FAPα foci in the stroma were formed only by the 7th day after bleomycin administration (Figure 4(7d) and Figure S5(7d)). Already by the 14th day, small αSMA+ fibrotic foci and cords had formed. Surprisingly, over half of the cells (55.5 ± 6.3%) were double-positive αSMA+FAPα+ (Figure 4A(14d),C and Figure S5(14d)). It is interesting to note that there were single perivascular αSMA+ zones in which FAPα expression was completely absent, which may indicate an alternative origin of myofibroblasts in these areas (Figure 5, (14d_focus *w*/*o* FAPα)).

On the 28th day after bleomycin administration, established αSMA+ fibrotic foci were visible in the lung tissue, with FAPα+ cells preserved mainly at the periphery similarly to CD90+ cells. Only in the zones of “ongoing fibrosis” did the relative positions of cell types remain consistent with the 14th day of observation (Figure 4(28d) and Figure S5(28d)). Based on these findings, we can assume that myofibroblast differentiation processes are active there. After 3 and 4 months, the number of αSMA+ areas significantly decreased, and were localized in the remaining foci, or in the form of single cells in the septa (Figure 4(3m,4m) and Figure S5(3m,4m)). After 5 months, αSMA expression was completely absent, which may indicate the completion of the fibrosis process.

One of the most important functions of myofibroblasts and their precursor is remodeling of the ECM by synthesizing its components. Therefore, we checked the location of fibronectin, one of the key profibrogenic proteins [25], around subpopulations of αSMA+ and FAPα+ cells at the stages of fibrotic foci formation (Figure S6). We showed that, according to visual assessment, fibronectin fibrils are located around FAPα+ or FAPα+ αSMA+ cells at the 14-day stage. At the same time, in the zone of formed fibrosis, especially in the obliteration zone, fibronectin is mainly deposited in zones rich in αSMA cells. Thus, it can be assumed that subpopulations of αSMA+FAPα+ cells play an important role in the deposition of the profibrogenic matrix at the initial formation stage of profibrotic foci.

### 3.5. Macrophages Colocalize with Activated FAPα+ Cells

Macrophages are highly plastic cells, represented in tissues by several phenotypes that transform into each other. It has been shown that M2-type macrophages can contribute to the maintenance of chronic inflammation in tissues and initiate the activation of stromal cells, which leads to the aggravation of fibrosis. To study possible mutual effects, we analyzed the dynamics of CD206+/CD163+ macrophage distribution and colocalization with FAPα+ cells in mouse lungs after bleomycin administration.

Thus, CD163+ macrophages in intact lungs were mostly localized around the walls of the bronchioles and large blood vessels; single cells were localized in the subpleural region. After the introduction of bleomycin, the number of these cells slightly increased on days 3 and 7. On day 14, local areas with CD163+ macrophages in connective tissue could be found; presumably, these were macrophages that had migrated from the wall of the bronchioles into the area of growing stroma. However, there was no pronounced correlation between the mutual localization of CD163+ and FAPα+ cells (Figure S7).

CD206 was revealed on alveolar macrophages, and the number of CD206+ cells increased by day 7 after bleomycin administration and remained stably high up to and including on day 28. Moreover, we observed a lower number of double-positive CD206+FAPα+ cells on day 28. Interestingly, the areas of FAPα+ cells were often framed by macrophages, which may indicate their mutual influence on each other (Figure 5 and Figure S8). During the fibrosis reversion phase, macrophages were almost completely absent in the lungs 3 months after bleomycin administration, which may indicate an insignificant contribution of these cells to the remodeling of connective tissue.

## 4. Discussion

The key morphofunctional unit of many progressive forms of fibrosis is a fibrotic focus, which is often considered a structure that cannot remodel. In this study, we tried to trace the stages of development and the destruction of fibrous tissue in lungs and focus on the one of the main cellular participants in developing fibrosis—stromal cells [3,26].

For many years, there has been an assumption that the initiators of the development of a fibrotic focus are immune cells that first to respond to tissue damage [27,28]. However, back in the 1990s, an alternative suggestion was made that stromal cells, rather than inflammatory cells, play an important role in initiating the fibrogenic response [29,30]. Thus, a number of studies have shown that stromal cells, which are called mesenchymal stromal cells, fibroblasts, or myofibroblasts in different sources, are localized near the damaged basement membrane of the epithelium and close the defects with both their “body” and the secreted ECM—this process corresponds to the beginning of the focus formation [12,31,32]. The exact phenotype of these “initiator” stromal cells has not been established. However, in 2001, FAPα+ activated stromal cells were discovered. It has been shown that these cells are the first to appear after injury and were localized near the damaged basal membrane of the epithelium in IPF [33]. In addition to secreting ECM proteins, FAPα+ cells in fibrosis also have many other functions including regulation of the immune system, increased angiogenesis, and participation in the differentiation of fibroblasts into myofibroblasts [10]. Accordingly, a number of studies indicate a close relationship between FAPα+ cells and the progression of the fibrotic process [34].

In this study, using the well-established model of murine bleomycin-induced pulmonary fibrosis, we have shown that FAPα+ cells, but not M2 macrophages, are observed in lung tissue on day 3 and 7 after injury. Thus, FAPα+ cells, along with cells of the immune system, may already be actively involved in the initiation of fibrotic changes at the earliest stages of fibrosis development, at least due to two mechanisms. Firstly, we have revealed that FAPα+ is expressed in more than half of the αSMA+ cells at the early stages of fibrosis, which may indicate their role in the differentiation of stromal cells into myofibroblasts. The involvement of FAPα in myofibroblast differentiation has long been discussed. Thus, there are studies that demonstrate that FAPα and αSMA are expressed on different cell subpopulations in vivo, and also that FAPα knockout does not affect the appearance of αSMA+ cells during fibrosis induction [34,35]. However, numerous studies suggest that increased FAPα expression can induce the differentiation of stromal cells into myofibroblasts [17,36,37]. Our data highlight that the overwhelming majority of myofibroblasts in bleomycin-induced pulmonary fibrosis in mice are FAPα+. At the same time, the presence of αSMA+FAPα− foci indicates that differentiation into myofibroblasts can also occur without the participation of FAPα. We have also shown that FAPα+ cells localize near fibronectin-rich areas during the formation of fibrotic foci. Our data are consistent with a number of literature data describing an increase in the expression of RNA of ECM proteins, as well as the colocalization of FAPα+ cells with collagen, decorin, and fibronectin in vivo [38,39,40,41]. It is known that fibronectin is one of the key proteins involved in the differentiation of fibroblasts into myofibroblasts [25,42,43]. Thus, it can be assumed that FAPα+ cells may promote further differentiation into myofibroblasts by changing the microenvironment. Since we have found subpopulations of FAPα cells with different localization and co-expression to other profibrotic markers, further study of their exact role at different stages seems to be a promising object for investigating the exact mechanisms regulating fibrosis. The second mechanism by which FAPα+ subpopulations can already contribute to the fibrosis progression at early stages is their high proliferative activity, which we have demonstrated by PCNA labeling. At the moment, there are only preliminary data that show the FAPα protein affects cell proliferation [34,44], so this issue still needs to be sorted out.

We also assessed in detail the dynamics of fibrotic foci development. When assessing the formation of the foci structure, it was shown that FAPα+ cells had already appeared on the third day after injury. Not later than the 7th day, cells of all types appeared—FAPα−αSMA+, FAPα+αSMA−, and FAPα+αSMA+, which at this stage formed alveolar wall thickenings and early foci. In parallel, the perivascular zones of CD90+ cells appeared. Already at the 14-days stage, a distinct formation of FAPα+αSMA+ foci was visible, in which FAPα+αSMA− cells formed on the periphery. At 28 days after the introduction of bleomycin, formed αSMA+ fibrotic foci were visible, in which FAPα+ cells were preserved mostly on the periphery, as well as CD90+ cells. Such a distribution of cells confirms the existing theory of a non-remodeling myofibroblastic core in the fibrotic focus, which is surrounded by a zone of actively differentiating cells that continue to pathologically remodel the tissue. Interestingly, similar dynamics—early appearance and then a sharp increase in the number of FAPα+ cells and then an increase in the number of myofibroblasts—were recently described in a model of toxic liver fibrosis in rats [45].

Since FAPα+ cells persist for a long time at the stages of fibrosis resolution, more than 3 months after bleomycin administration, it can be assumed that they are also involved in the remodeling of fibrotic tissue. However, the question of how the FAPα function is “switched” from profibrotic to reparative remains open. A possible answer may be the presence of different subpopulations of FAPα+ cells and a change in the FAPα functioning depending on its protein partners. Thus, the literature has shown the presence of FAPα+CD90+ and FAPα+CD90− in inflammatory diseases, where FAPα+CD90− act as matrix-destroying cells or cells that regulate this process, while FAPα+CD90+ exhibit immunomodulatory properties [44,46]. Accordingly, animals knocked out by CD90 show a worsening development of fibrosis [47,48,49], which once again emphasizes the importance of this protein not so much as a marker of the general fibroblast population but mostly as one that identifies specialized stromal cell subtypes. The hypothesis of the important contribution of specific subpopulations of stromal cells into the fibrosis resolution is strongly supported in our study by the revealed structures formed by CD90+ and CD90+ FAPα+ cells in recovering lung tissue, which we have called “remodeling foci”.

Type-2 macrophages are considered anti-inflammatory, and we observed an increase in the number of CD206+ macrophages in the lungs at 14 and 28 days after bleomycin injury and migration of CD163+ macrophages from the peribronchial areas into the connective tissue, which may indicate a transition to the tissue remodeling stage at these times. The persistence of M2 macrophages in the tissue may support the number of activated FAP+ cells, but further studies are required to determine the functional differences between FAP+ cells early after injury and late remodeling. The resolution of fibrosis at 3 months and later correlates with a decrease in the number of CD206+ macrophages.

## 5. Conclusions

Our findings indicate that activated stromal cells, capable of rapid proliferation and ECM remodeling, can be considered as a promising new target population in the fight against fibrosis, instead of myofibroblasts. For a long time, myofibroblasts were recognized as the key cell type mediating the development of fibrosis and, thus, were the main targets for therapeutic agents. However, approaches based on the suppression of myofibroblast function or stimulation of their elimination have not demonstrated sufficient effectiveness in clinical practice. Accumulating data on the multiple functions of FAPα allows us to assume that this protein is not only a marker of the activated state of stromal cells in various pathologies but also contributes to the progression of fibrosis. FAPα expression, as we have shown, appears incredibly early in damaged lungs, and apparently not only triggers the profibrotic response of the stroma, but also participates in supporting the differentiation of activated cells into myofibroblasts, as well as attracting immune cells. However, the possible role of FAPα in the reversion of fibrosis leaves open the question of finding more subtle mechanisms for its regulation, as well as searching for specific functions of FAPα+ cell subpopulations.

## Figures and Tables

**Table 1:** List of primary antibodies used in the work.

Antigen	Company	Catalog Number
CD90 (Thy1)	Invitrogen	PA5-80127
αSMA	BioLegend (San Diego, CA, USA)	904601
cytokeratin	abcam	ab9377
CD206	abcam	ab64693
CD163	abcam	ab182422
PCNA	ThermoFisher (Waltham, MA, USA)	PA5-27214
fibronectin	abcam	ab2413

## Data Availability

The original contributions presented in this study are included in the article/Supplementary Material. Further inquiries can be directed to the corresponding author.

## Supplementary Materials

The following supporting information can be downloaded at https://www.mdpi.com/article/10.3390/cells13242064/s1, Figure S1: Quantification of dynamic changes in the lung tissue density measured via MRI between 28 days and 3 months after single bleomycin instillation; Figure S2: Remodeling foci are formed by the stage of active resolution of fibrosis (4 months after the introduction of bleomycin). Representative images; H&E; 14 (14d), 28 (28d) days and 3 (3m) or 4 (4m) months after bleomycin instillation; Figure S3: Enlarged images of FAPa+ cells, 3 days after bleomycin instillation. Pancytokeratin (green), FAPα (purple), DAPI (blue). PCNA (green), FAPα (red), DAPI (gray). Scale bar = 100 μm; Figure S4: The areas, occupied by FAPα cells, are adjacent to the remaining alveolar lumens on day 28. CD90 (yellow), FAPα (magenta), DAPI (blue). Scale bar = 100 μm; Figure S5: Dynamic of fibrotic foci formation and remodeling in the model of bleomycin-induced pulmonary fibrosis in mice. Representative image; Intact group (int); remodeling foci (rf); 3 (3d), 7 (7d), 14 (14d), 28 (28d) days and 3 (3m) or 4 (4m; 4m_rf—remodeling foci) months after bleomycin instillation. αSMA (cyan), CD90 (yellow), FAPα (magenta), DAPI (gray). Scale bar = 100 μm; Figure S6: Fibronectin deposition in the model of bleomycin-induced pulmonary fibrosis in mice. Representative image; 14 (14d) and 28 (28d) days after bleomycin instillation. αSMA (cyan), fibronectin (yellow), FAPα (purple), DAPI (gray). Scale bar = 100 μm; Figure S7: Localization of CD163 and FAPα + cells. Representative images. Representative image; Intact group (int); 7 (7d) and 14 (14d) days after bleomycin instillation. CD163 (cyan), FAPα (magenta), DAPI (gray). Scale bar = 100 μm; Figure S8: Areas occupied by CD206+ macrophages and FAPα+ cells are often adjacent to each other. Representative images, 28 days after bleomycin instillation. CD206 (cyan), FAPα (magenta), DAPI (gray). Scale bar = 100 μm; Figure S9: Immunocytochemical analysis for the αSMA, CD90, CD163, CD206, cytokeratin, PCNA and FAPα expressions in pulmonary tissue (antibody) compared with staining with the corresponding isotype control antibodies (IgG). Scale bar = 100 μm.
