# Supplementary material for: The Role of Activated Stromal Cells in Fibrotic Foci Formation and Reversion
Source: Cells. 2024 Dec 13;13(24):2064. doi: 10.3390/cells13242064 (PMC11674712; doi:10.3390/cells13242064)
Supplement: Supplementary file 1 [file cells-13-02064-s001.zip › cells-3312780-SI.pdf]

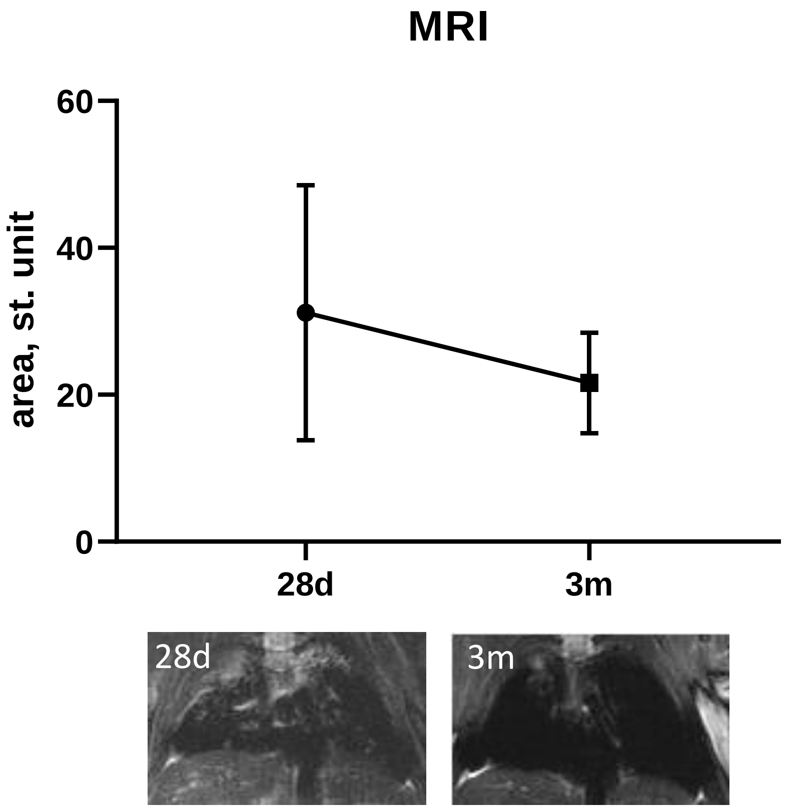

Figure S1. Quantification of dynamic changes in the lung tissue density measured via MRI between 28 days and 3 months after single bleomycin instillation

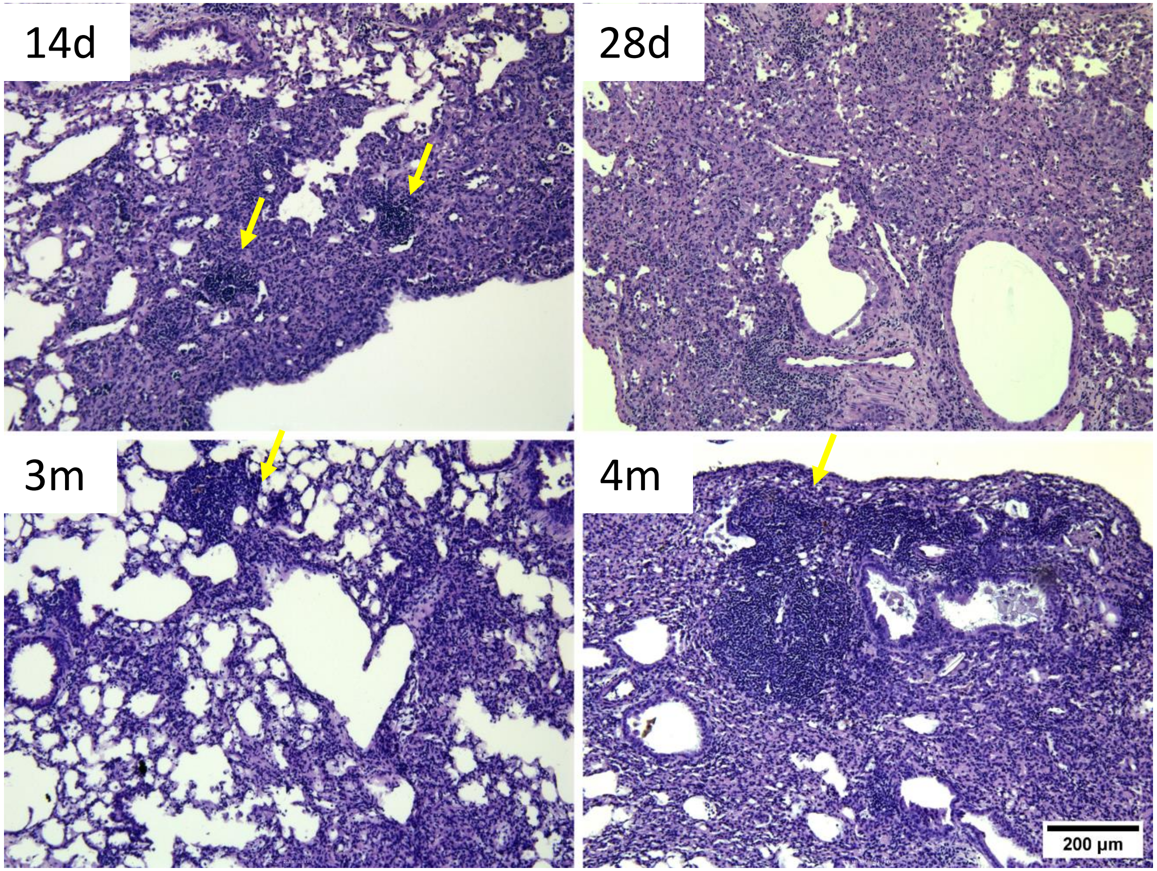

Figure S2. Remodeling foci are formed by the stage of active resolution of fibrosis (4 months after the introduction of bleomycin). Representative images; H&E; 14 (14d), 28 (28d) days and 3
(3m) or 4 (4m) months after bleomycin instillation. Yellow arrows point to field of remodeling foci.
Scale bar – 200 µm.

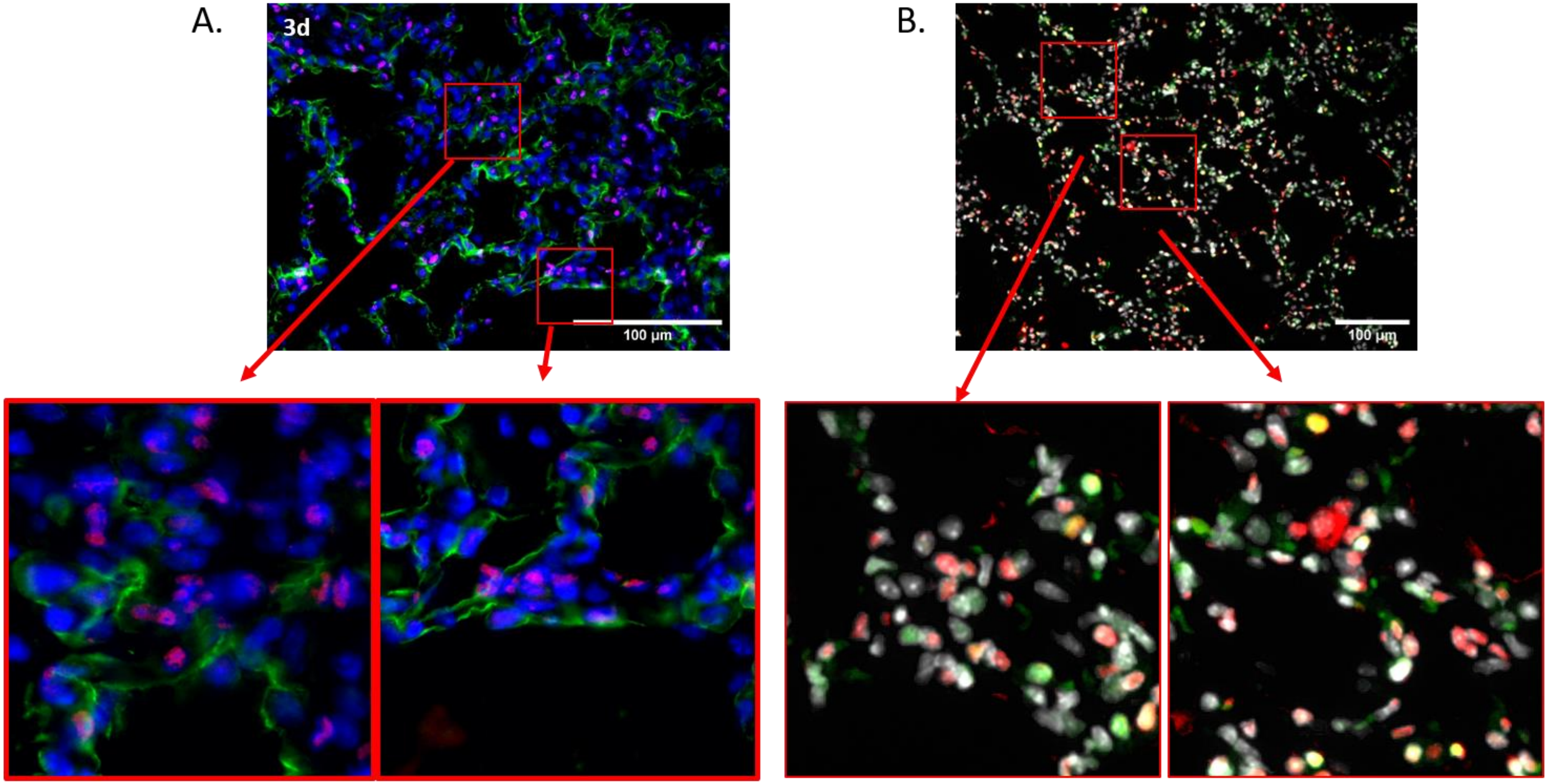

Figure S3: Enlarged images of FAPa+ cells, 3 days after bleomycin instillation.

A. Pancytokeratin (green), FAPα (purple), DAPI (blue).

B. PCNA (green), FAPα (red), DAPI (grey).

Scale bar = 100 µm

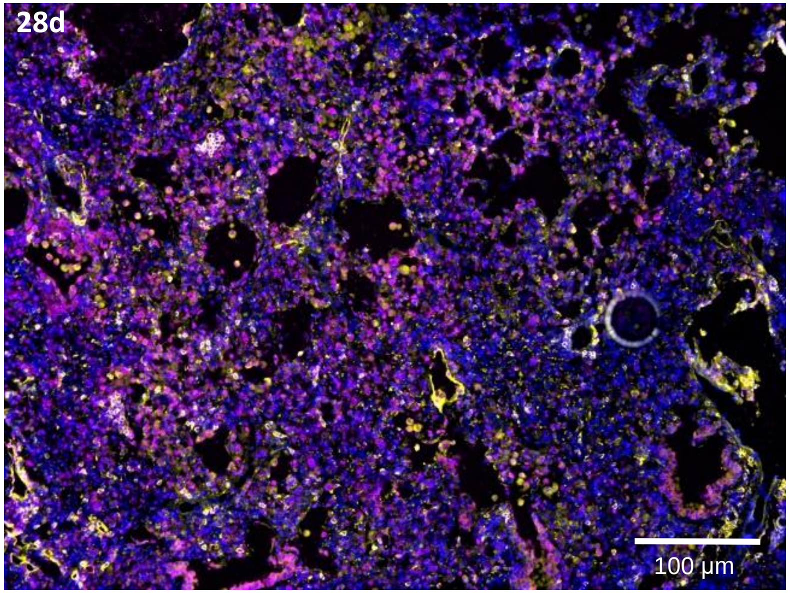

Figure S4. The areas, occupied by FAPα cells, are adjacent to the remaining alveolar lumens on day 28 after bleomycin instillation. CD90 (yellow), FAPα (purple), DAPI (blue). Scale bar = 100 µm

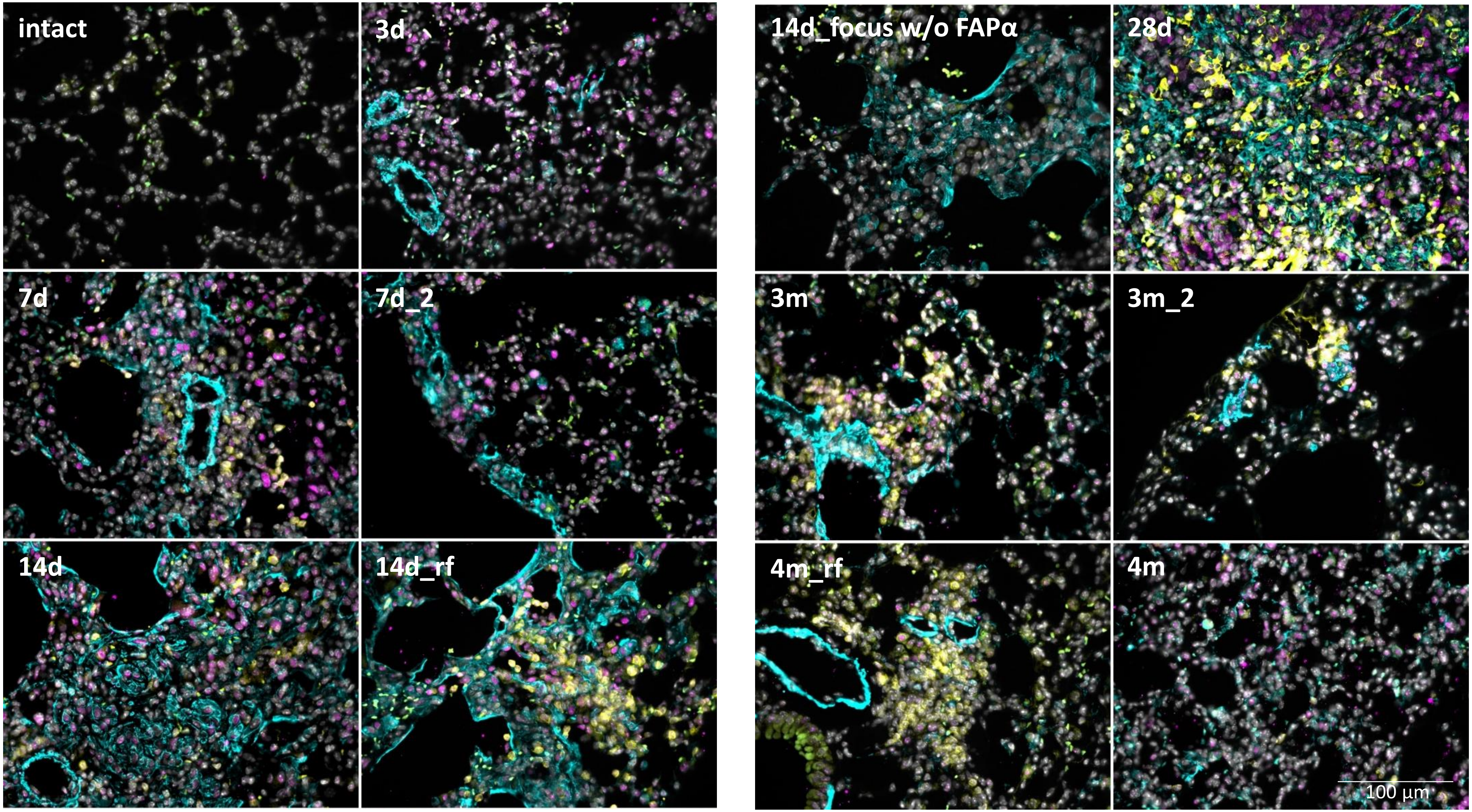

Figure S5: Dynamic of fibrotic foci formation and remodeling in the model of bleomycin-induced pulmonary fibrosis in mice. Representative image; Intact group (int); remodeling foci (rf); 3
(3d), 7 (7d), 14 (14d), 28 (28d) day and 3 (3m) or 4 (4m; 4m_rf - remodeling foci) month after bleomycin instillation. αSMA (cyan), CD90 (yellow), FAPα (purple), DAPI (grey). Scale bar = 100 µm

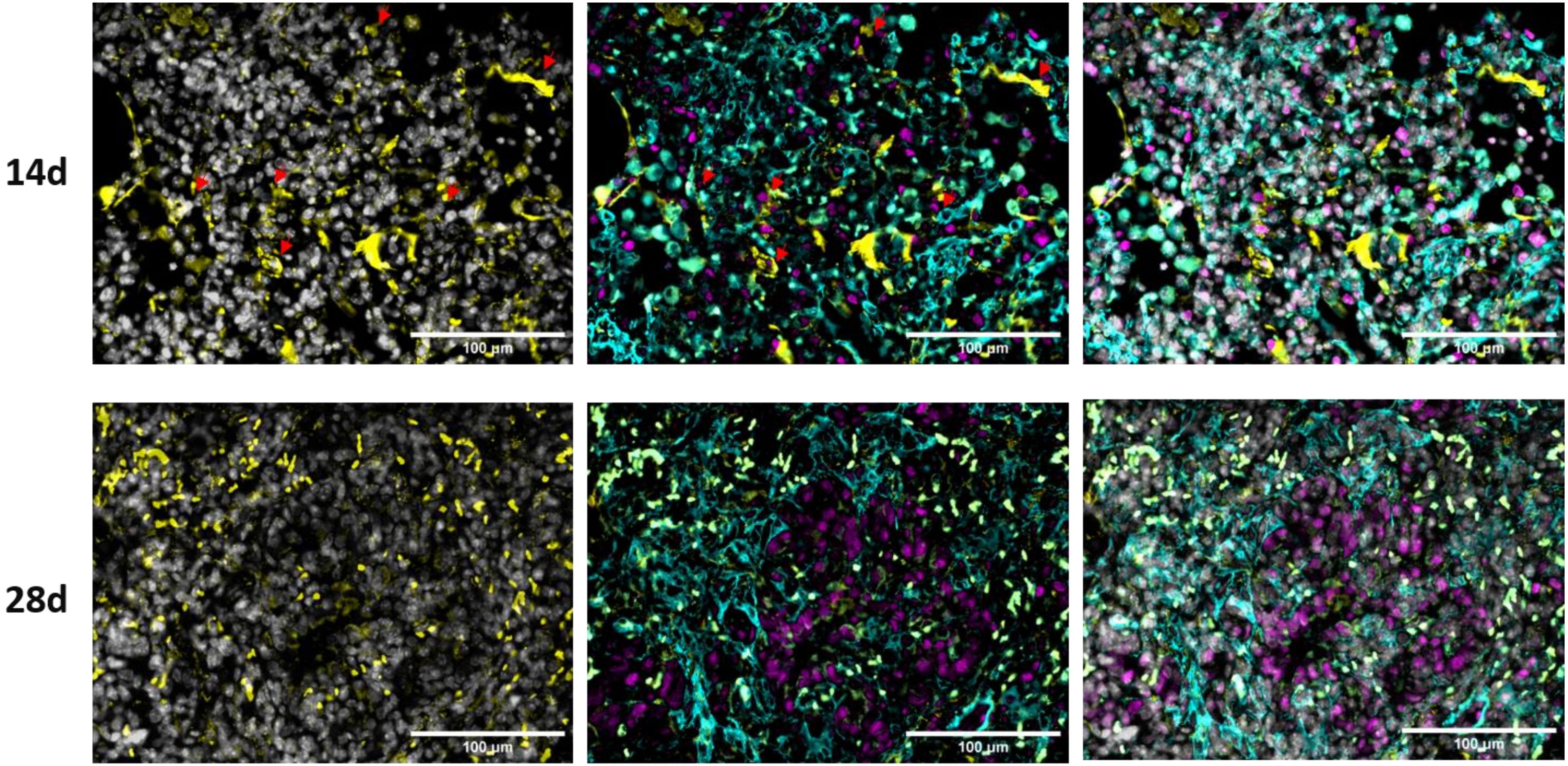

Figure S6: Fibronectin deposition in the model of bleomycin-induced pulmonary fibrosis in mice. Representative image; 14 (14d) and 28 (28d) days after bleomycin instillation. αSMA (cyan), fibronectin (yellow), FAPα (purple), DAPI (grey). Scale bar = 100 µm

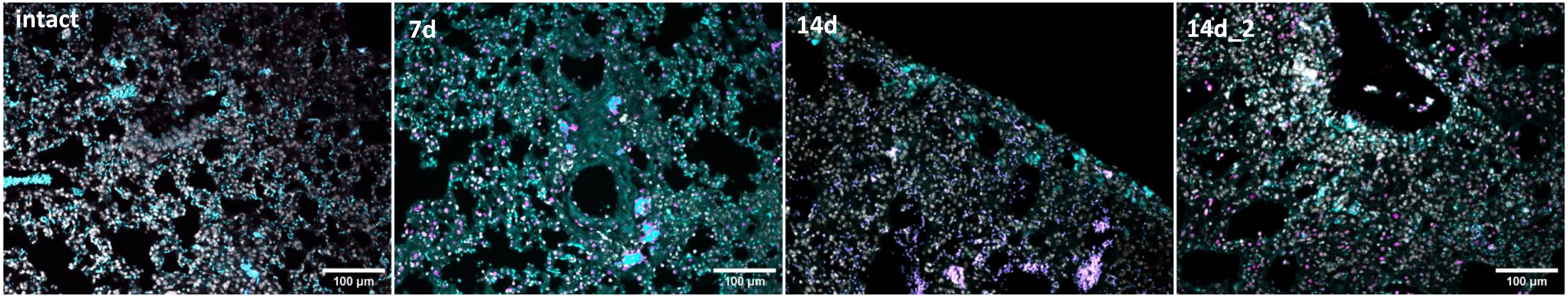

Figure S7 Localization of CD163 and FAPα + cells. Representative images. Representative image; Intact group (int); 7 (7d) and 14 (14d) after bleomycin instillation. CD163 (cyan), FAPα (purple), DAPI (grey). Scale bar = 100 µm

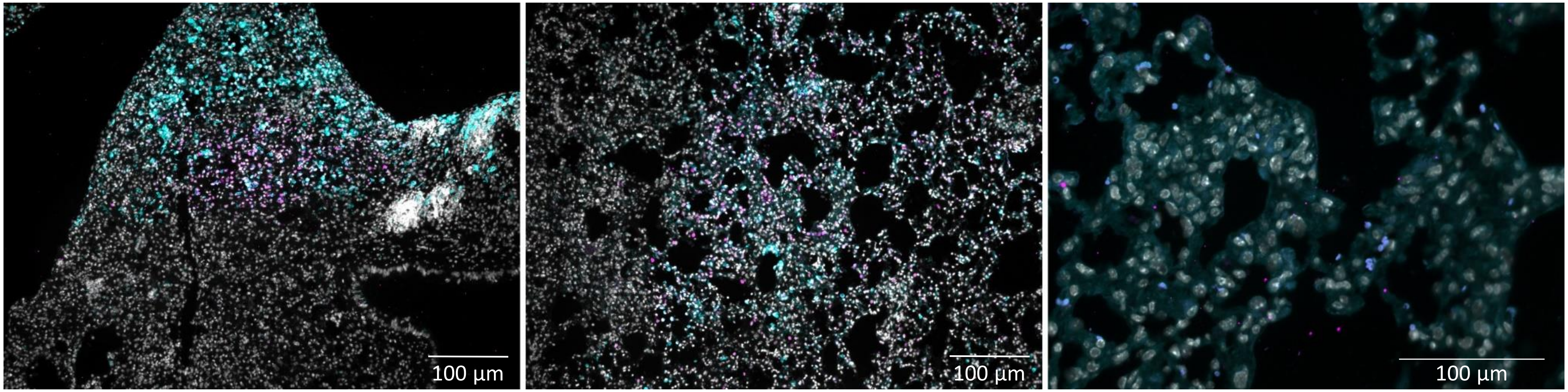

Figure S8. Areas occupied by CD206+ macrophages and FAPα + cells are often adjacent to each other. Representative images, 28 days after bleomycin instillation. CD206 (cyan), FAPα (purple), DAPI (grey).

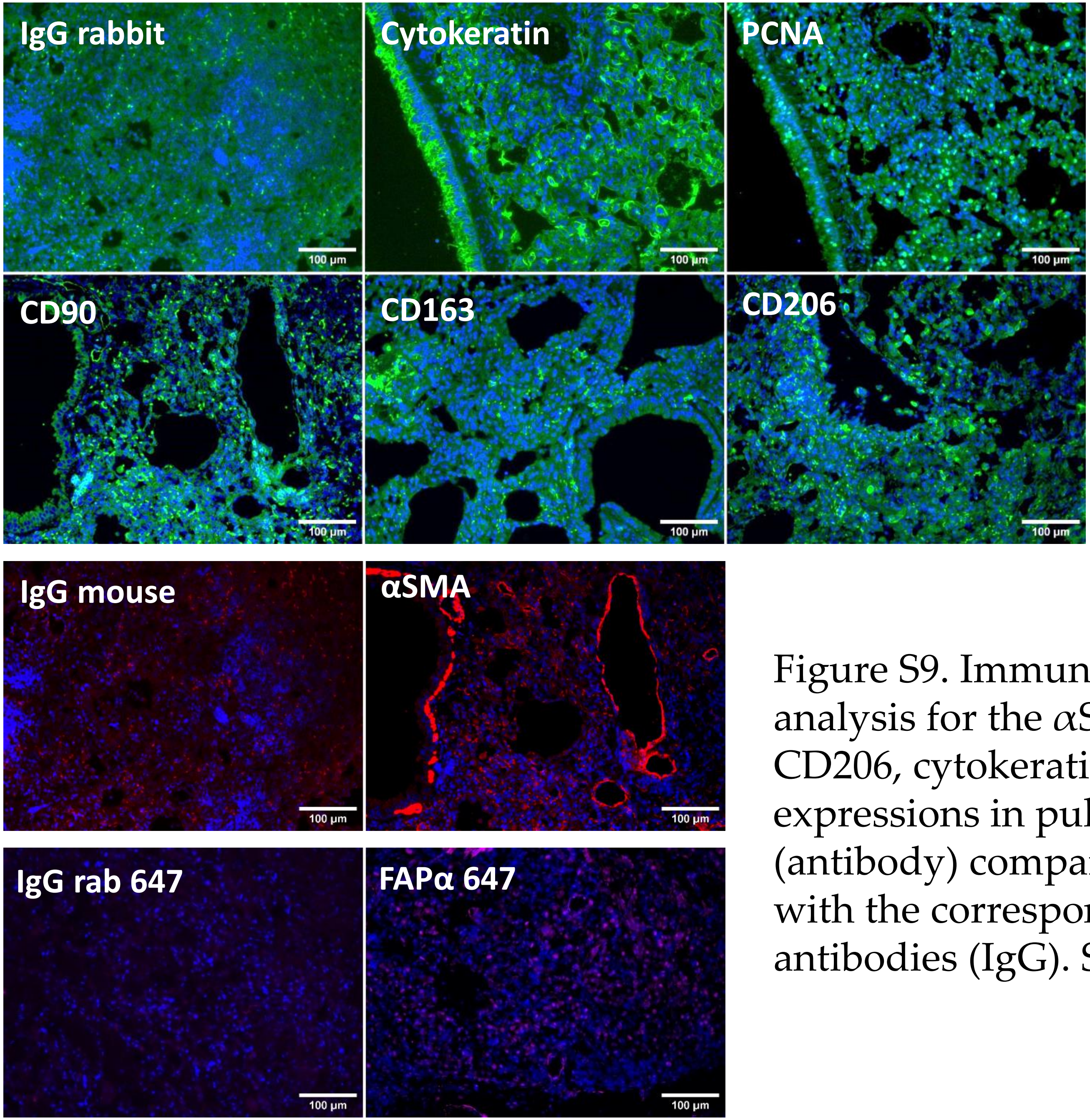

Figure S9. Immunocytochemical analysis for the αSMA, CD90, CD163, CD206, cytokeratin, PCNA and FAPα expressions in pulmonary tissue (antibody) compared with staining with the corresponding isotype control antibodies (IgG). Scale bar = 100 μm.
